# Propofol infusion syndrome: early blood purification to the rescue?

**DOI:** 10.1186/s13054-016-1364-8

**Published:** 2016-07-04

**Authors:** Patrick M. Honore, Herbert D. Spapen

**Affiliations:** ICU Department, Universitair Ziekenhuis Brussel, Vrije Universiteit Brussel, 101, Laarbeeklaan, 1090 Jette Brussels, Belgium

We read with interest the comprehensive review of Krajčová et al. [1] on propofol infusion syndrome (PRIS). Of particular importance is the high (51 %!) associated mortality which is mainly determined by cardiac failure, therapy-resistant bradyarrhythmia, metabolic acidosis, and rhabdomyolysis [1]. The authors provide a detailed insight on the pathophysiologic mechanism(s) underlying PRIS but do not discuss therapy, probably because therapeutic options are limited and merely supportive. Basically, propofol should be withdrawn immediately and adequate hemodynamic support must be ensured. Patients who develop rhabdomyolysis-associated acute kidney injury (AKI) are treated with hemodialysis or hemofiltration. Extracorporeal membrane oxygenation was life-saving in two patients [2]. A recent case report described rapid hemodynamic improvement and resolution of lactic acidosis after one single session of plasma exchange [3].

We suggest that early initiation of continuous renal replacement therapy (CRRT), even in the absence of well-established indications such as AKI, metabolic acidosis, or hyperkalemia, might add a survival benefit. Propofol undergoes hepatic metabolization and is rapidly cleared by the kidneys as propofol-glucuronide and 2,6-diisopropyl-1,4-quinol sulfo- and glucuro-conjugates [4]. Unlike the highly lipophilic parent drug, these toxic water-soluble metabolites can be eliminated by CRRT. Caution is needed, however, when implementing such a strategy. Prolonged PRIS-related cardiogenic shock with severely compromised hepatic and muscle perfusion may dramatically reduce mitochondrial citrate metabolism and result in citrate accumulation. As citrate is increasingly used for regional anticoagulation during CRRT, tight monitoring of the ionized/total calcium ratio is imperative to obviate metabolic complications related to citrate intoxication. A ratio exceeding 2.25 strongly suggests citrate overdose and requires replacing citrate by unfractionated heparin [5].

## Abbreviations

AKI, acute kidney injury; CRRT, continuous renal replacement therapy; PRIS, propofol infusion syndrome
